# An *Agrobacterium*‐delivered CRISPR/Cas9 system for high‐frequency targeted mutagenesis in maize

**DOI:** 10.1111/pbi.12611

**Published:** 2016-09-05

**Authors:** Si Nian Char, Anjanasree K. Neelakandan, Hartinio Nahampun, Bronwyn Frame, Marcy Main, Martin H. Spalding, Philip W. Becraft, Blake C. Meyers, Virginia Walbot, Kan Wang, Bing Yang

**Affiliations:** ^1^ Department of Genetics Development and Cell Biology Iowa State University Ames IA USA; ^2^ Department of Agronomy Iowa State University Ames IA USA; ^3^ Donald Danforth Plant Science Center St. Louis MO USA; ^4^ Department of Biology Stanford University Stanford CA USA

**Keywords:** *anthocyaninless*, *Argonaute*, CRISPR/Cas9, gene editing, maize, targeted mutagenesis

## Abstract

CRISPR/Cas9 is a powerful genome editing tool in many organisms, including a number of monocots and dicots. Although the design and application of CRISPR/Cas9 is simpler compared to other nuclease‐based genome editing tools, optimization requires the consideration of the DNA delivery and tissue regeneration methods for a particular species to achieve accuracy and efficiency. Here, we describe a public sector system, ISU Maize CRISPR, utilizing *Agrobacterium*‐delivered CRISPR/Cas9 for high‐frequency targeted mutagenesis in maize. This system consists of an *Escherichia coli* cloning vector and an *Agrobacterium* binary vector. It can be used to clone up to four guide RNAs for single or multiplex gene targeting. We evaluated this system for its mutagenesis frequency and heritability using four maize genes in two duplicated pairs: *Argonaute 18* (*ZmAgo18a* and *ZmAgo18b*) and *dihydroflavonol 4‐reductase* or *anthocyaninless* genes (*a1* and *a4*). T_0_ transgenic events carrying mono‐ or diallelic mutations of one locus and various combinations of allelic mutations of two loci occurred at rates over 70% mutants per transgenic events in both Hi‐II and B104 genotypes. Through genetic segregation, null segregants carrying only the desired mutant alleles without the CRISPR transgene could be generated in T_1_ progeny. Inheritance of an active CRISPR/Cas9 transgene leads to additional target‐specific mutations in subsequent generations. Duplex infection of immature embryos by mixing two individual *Agrobacterium* strains harbouring different Cas9/gRNA modules can be performed for improved cost efficiency. Together, the findings demonstrate that the ISU Maize CRISPR platform is an effective and robust tool to targeted mutagenesis in maize.

## Introduction

Clustered regularly interspaced short palindromic repeat/CRISPR‐associated Cas9 (CRISPR/Cas9) constitutes an adaptive immune system in many proteobacteria and archaea (Bhaya *et al*., 2011). These genes enable hosts to eliminate invading genetic parasites such as virus and plasmid DNA. Type I, II and III CRISPR/Cas systems with distinct characteristics of guide RNAs and Cas proteins have been documented. The Type II CRISPR/Cas system from *Streptococcus pyogenes* has been most widely adapted for site‐specific genomic alteration or genome editing. Other CRISPR/Cas systems such as those derived from *Neisseria meningitidis* and *Streptococcus thermophiles* also have been adapted for genome editing in mammals (Hou *et al*., 2013; Xu *et al*., 2015). Modified CRISPR/Cas9 systems suitable for eukaryotes consist of a nuclear localized endonuclease and a guide RNA; this complex is referred to here as Cas9/gRNA. Unlike the predecessor zinc finger nuclease (ZFN) and TAL effector nuclease (TALEN), which involve dimerizing fusion proteins including the DNA binding domains of ZF and TAL and cleavage domains of *Fok*I endonuclease, Cas9/gRNA is a ribonucleoprotein active on target DNA. gRNA is a chimeric molecule of CRISPR RNA (crRNA) and transactivating crRNA (tracrRNA) preceded by a spacer sequence of 18–20 nucleotides complementary to the target DNA. Cas9 contains both RuvC and HNH DNA cleavage domains that cause DNA double‐strand breaks (DSB) predominantly located 3 bp upstream of the protospacer adjacent motif (PAM) sequence (5′‐NGG or 5′‐NAG for *S. pyogenes* Cas9) of the target DNA. Subsequently, host DSB DNA repair *in vivo* utilizes error‐prone nonhomologous end‐joining (NHEJ) or homology‐directed repair (HDR). NHEJ often leads to random DNA insertions or deletions (indel mutations) at the cleavage site (so‐called targeted mutagenesis), while HDR can be exploited for precise sequence or gene replacement or insertion by providing a donor DNA template with sequence homology to the predicted DSB region.

Different Cas9/gRNA systems have been tailored for targeted genomic alterations in both prokaryotes and eukaryotes. Tailored Cas9/gRNA systems have been successfully deployed into plants as DNA to generate site‐specific mutagenesis in both monocotyledonous and dicotyledonous species including Arabidopsis (Feng *et al*., 2014; Jiang *et al*., 2014), tomato (Brooks *et al*., 2014; Cermak *et al*., 2015), potato (Wang *et al*., 2015), soybean (Li *et al*., 2015), rice (Feng *et al*., 2013; Zhou *et al*., 2014), sorghum (Jiang *et al*., 2013), wheat (Wang *et al*., 2014), barley (Lawrenson *et al*., 2015) and maize (Liang *et al*., 2014; Xing *et al*., 2014; Svitashev *et al*., 2015). The Cas9/gRNA can also be delivered into protoplasts of Arabidopsis, tobacco, lettuce and rice as a protein/RNA complex (a ribonucleoprotein, RNP) to induce mutations in cells, some of which can be regenerated into a gene‐mutated plant (Woo *et al*., 2015).

Although the CRISPR technology is simpler than ZFNs or TALENs, it must be optimized for each plant species to accommodate the type of tissue and the transformation delivery method. For example, different RNA polymerase II‐based promoters (ubiquitin gene promoters, viral CaMV 35S promoter, etc.) suitable for the expression of Cas9, or RNA polymerase III‐dependent promoters (U3, U6, etc.) for driving gRNA expression, need to be tested for efficacy in specific plant species. For DNA delivery, there are two major transformation methods: *Agrobacterium tumefaciens*‐mediated or biolistic (gene gun)‐mediated methods. Both methods are effective in transforming plant species that are not amenable to regeneration from single‐cell protoplasts. The *Agrobacterium*‐mediated method is more popular, because it has a propensity to insert single or a low copy number of transgenes and does not require an expensive particle gun apparatus and supplies.

Maize (*Zea mays*) supplies 25% of the world's calories. Genome editing protocols for this crop have been developed, including the application of ZFNs (Shukla *et al*., 2009), TALENs (Char *et al*., 2015) and Cas9/gRNA (Feng *et al*., 2016; Liang *et al*., 2014; Svitashev *et al*., 2015; Xing *et al*., 2014; Zhu *et al*., 2016). The first report of Cas9/gRNA maize mutagenesis was by Liang *et al*. (2014) in protoplasts using polyethylene glycol (PEG) to mediate DNA uptake. By using the maize U3 promoter for gRNA and the CaMV 35S promoter for a rice codon‐optimized *Cas9*, Liang *et al*. (2014) achieved 16.4% mutation frequency for one gRNA and 19.1% for the second gRNA in experiments targeting the *inositol phosphate kinase* gene (*ZmIPK*) in mesophyll protoplasts. Similarly, Xing *et al*. (2014) built a suite of Cas9/gRNA vectors; for maize, the Cas9/gRNA construct consisted of a maize codon‐optimized *Cas9* under the maize *ubiquitin 1* gene promoter and gRNA under the rice U3 or wheat U3 promoters. A construct targeting *ZmHKT* was delivered by *Agrobacterium* into immature embryos of B73; twenty T_0_ plants showed mutations of *ZmHKT*, although no explicit frequency was reported (Xing *et al*., 2014). Svitashev *et al*. (2015) also reported CRISPR/Cas9‐induced mutagenesis in maize, as well as gene replacement and gene insertion using biolistic‐mediated transformation. The two key reagents of their protocol were the maize *ubiquitin 1* gene promoter joined to a maize codon‐optimized *Cas9*, and a maize U6 promoter for gRNAs. A mixture of *Cas9*, gRNAs (in either the DNA gene or RNA form of gRNA), plus transformation selection and visual marker genes were co‐bombarded into immature embryos of the maize Hi‐II genotype. Additionally, *in vitro* transcribed guide RNAs were introduced into embryos expressing a stably integrated *Cas9* transgene (Svitashev *et al*., 2015). Most recently, two groups demonstrated the feasibility of *Agrobacterium*‐delivered Cas9/gRNA in targeted mutagenesis of the endogenous *phytoene synthase* at 13% frequency (Zhu *et al*., 2016) or *Zmzb7* at 2% frequency (Feng *et al*., 2016) assessed in T_0_ Hi‐II plants.

In this work, we present an easy‐to‐use binary vector system, ISU Maize CRISPR, for efficient site‐specific mutagenesis in maize using *Agrobacterium*‐mediated maize transformation. Our intention is to provide the public research community with an enabling platform for maize genome editing. We validated the Cas9/gRNA and *Agrobacterium*‐mediated protocol using two maize gene families, *Argonaute 18* and *dihydroflavonol 4‐reductase*. For each gene family, with members on two different chromosomes, we designed two gRNAs to target two sites within each allele. Here, we show that this vector can be used to insert up to four gRNAs for single or multiplex mutagenesis. We also show that the *Agrobacterium* binary vector system achieves highly efficient and heritable site‐specific mutagenesis for both maize hybrid genotype Hi‐II and inbred B104. Because the preparation of staged maize embryos can be rate limiting and maize transformation process can be costly, we also demonstrated that efficient mutagenesis can be achieved by mixing two *Agrobacterium* strains for one infection experiment to generate transgenic plants independently mutated in each target by separate Cas9/gRNA construct. We confirm that the continuous presence of Cas9/gRNA in transgenic maize can cause mutagenesis of target genes of interest in subsequent generations. The Cas9/gRNA transgenic lines, therefore, can be used to convey the CRISPR‐based mutagenesis by genetic cross to maize lines that are not amenable to genetic transformation.

## Results

### Targeted mutagenesis strategy

**Figure 1:**
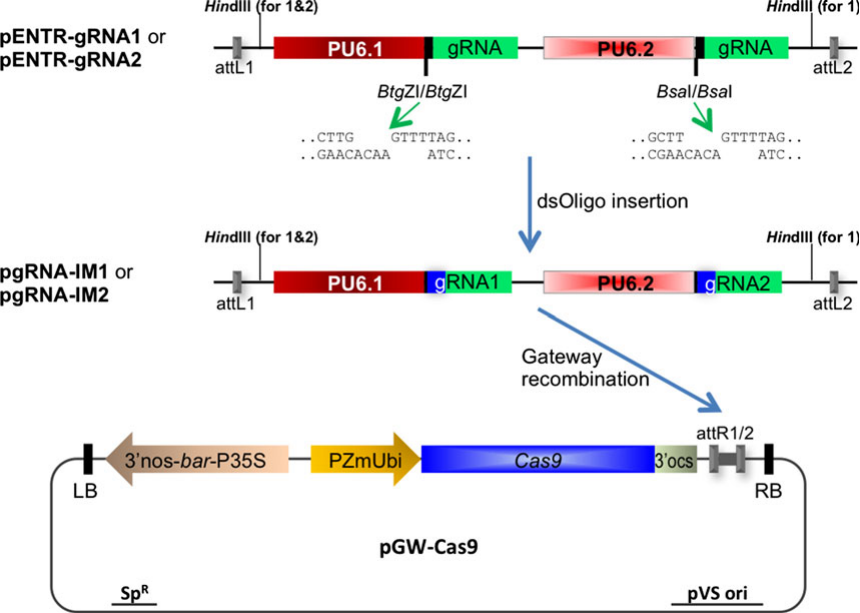
Schematic diagram of Cas9/gRNA construction. Cloning vectors pENTR‐gRNA1 (with two *Hin*dIII sites) or pENTR‐gRNA2 (with one *Hin*dIII site) were sequentially digested with *Btg* ZI and *Bsa*I restriction enzymes for the insertions of two double‐stranded oligonucleotides. The subcloning resulted in two intermediate constructs, pgRNA‐IM1 and pgRNA‐IM2, each carrying two gRNA expression cassettes. The cassettes flanked by the Gateway recombination sequences attL1 and attL2 were mobilized to the binary vector pGW‐Cas9 through Gateway recombination, resulting in a single plasmid Cas9/gRNA binary construct for *Agrobacterium*‐mediated gene transfer.

A schematic of the ISU Maize CRISPR plasmids used for *Agrobacterium*‐mediated Cas9/gRNA introduction into maize is shown in Figure 1. The gRNA vectors are based on pENTR‐gRNA1 and pENTR‐gRNA2 described previously (Zhou *et al*., 2014). In each intermediate vector, two different rice U6 small nuclear RNA gene promoters (PU6.1 and PU6.2) are used to express the gRNA genes. The first gRNA scaffold (85 nucleotides) is preceded by a cloning site containing two *Btg*ZI sites in a tail‐to‐tail orientation downstream of PU6.1. The second gRNA scaffold follows a pair of tail‐to‐tail‐oriented *Bsa*I sequences downstream of PU6.2. Two sequential rounds of cloning permit the insertion of custom double‐stranded gRNA spacer DNA sequences into these double *Btg*Z1 and double *Bsa*1 restriction enzyme sites in the vectors to generate intermediate constructs pgRNA‐IM1 or pgRNA‐IM2 (Figure 1).

As described in an earlier publication (Zhou *et al*., 2014), these two vectors differ by one feature: pENTR‐gRNA1 possesses two *Hin*dIII sites near the Gateway recombination sites attL1 and attL2, while pENTR‐gRNA2 has only one *Hin*dIII site near the attL1 site (Figure 1). This feature allows pgRNA‐IM2 to receive the gRNA cassettes from pgRNA‐IM1 via *Hin*dIII digestion and subcloning. Therefore, this strategy can be used to construct up to four gRNAs, simultaneously targeting up to four DNA sequences in the maize genome.

The guide RNA spacer sequences were designed based on the maize B73 reference genome sequence (Schnable *et al*., 2009) using the CRISPR Genome Analysis Tool (Brazelton *et al*., 2015; http://cbc.gdcb.iastate.edu/cgat/). The relevant target regions in Hi‐II and B104 genotypes were PCR‐amplified and confirmed by sequencing. All pgRNA‐IM constructs were confirmed for sequence accuracy at the insertion sites and flanking regions by Sanger sequencing. The confirmed gRNA cassette can be mobilized through Gateway recombination to the destination vector pGW‐Cas9. The vector is built on the backbone of pMCG1005 (a gift from Dr. Vicki Chandler); this vector contains a rice codon‐optimized *Cas9* with the maize *ubiquitin 1* gene promoter and the *bar* gene with a 4× CaMV 35S promoter used as transformation selectable marker (Figure 1). The binary plasmid is mobilized into *Agrobacterium* strain EHA101 for the transformation of maize immature embryos.

**Figure 2:**
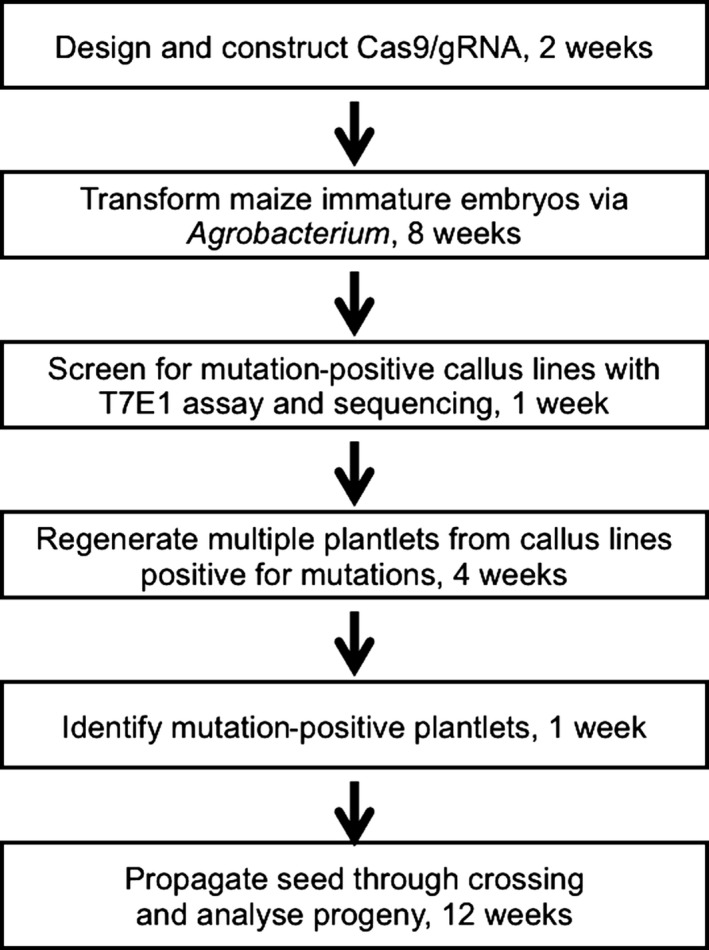
A flow chart of targeted mutagenesis in maize using *Agrobacterium*‐mediated transformation illustrates the main steps in CRISPR‐based mutagenesis. A minimum of 7 months is required from embryo transformation to production of mutant seeds.

*Agrobacterium‐*based maize transformation was previously described (Frame *et al*., 2006). For a typical site‐directed mutagenesis project, 20–30 bialaphos‐resistant callus lines are identified for genotyping using the T7 endonuclease I (T7E1) assay (Char *et al*., 2015). This assay uses PCR amplification of the target gene followed by melting and reannealing the PCR products; homozygous individuals (two copies of the wild‐type or two copies of the same mutant allele) yield a single duplex, while heterozygous individuals (diallelic mutants or wild‐type allele plus mutated allele) yield multiple duplexes containing mismatches. The mismatched bases are targets for T7E1 cleavage, resulting in multiple fragments resolved by agarose gel electrophoresis. Comparison of restriction fragment patterns to wild‐type size standards permits the classification of lines as diallelic mutants (DA), monoallelic mutants (MA) or nonmutant lines, with subsequent Sanger sequencing used to precisely describe the mutations (Char *et al*., 2015). Typically, ten independent callus lines with defined mutations are selected for plant regeneration, followed by self‐crossing or crossing to a wild‐type line to produce transgenic seeds. Multiple (usually two to five) plantlets are produced from each callus line. During T_0_ growth, DNA from leaf samples are subjected to the T7E1 assay and sequencing of the site‐specific PCR amplicons. The time frame from construct design to seed from a CRISPR maize line is approximately 7 months (Figure 2).

### Cas9/gRNA constructs induce highly efficient mutagenesis in four genes

**Figure 3:**
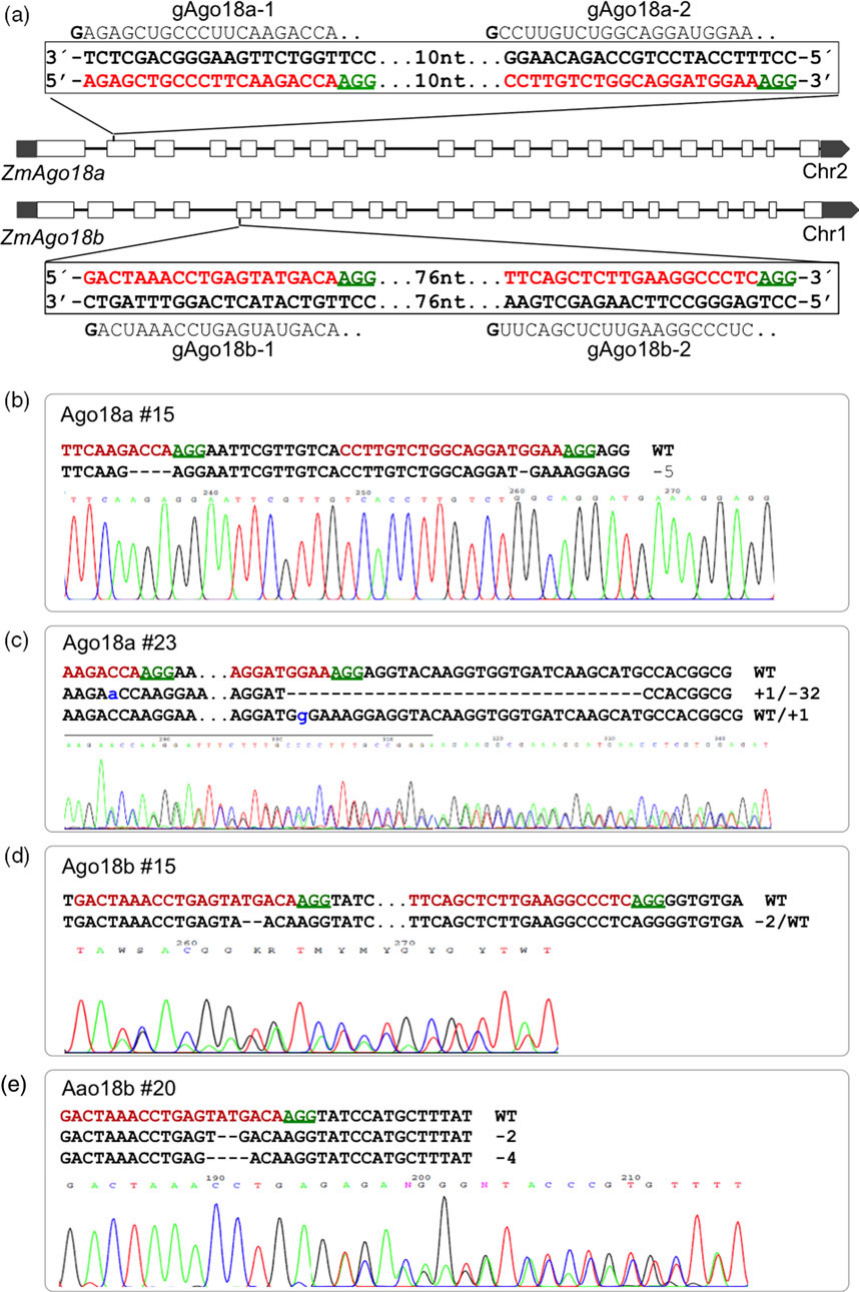
Cas9/gRNA‐induced mutations in *ZmAgo18a* and *ZmAgo18b*. (a) Structure of the paralogous *Ago18* genes present on chromosomes 1 and 2 and the gRNAs designed to generate DSBs in exons (blank bars). gRNAs, gAgo18a‐1 and gAgo18a‐2 (above the double‐strand box for *ZmAgo18a*), and gAgo18b‐1 and gAgo18b‐2 (below the double‐strand box for *ZmAgo18b*). Nucleotides in red represent target sites, and green underlined nucleotides indicate PAM sequences for the gRNAs. 10 and 76 nt represent the numbers of nucleotides between the two target sites in each gene. (b–e) Sequences from selected T_0_ plants with site‐specific mutations accompanied by corresponding regions of the sequencing chromatograms. The nucleotide changes (dashes for deletion, lowercase letter for insertion and WT for unaltered) are also indicated to the right side of each sequence. Dots in Ago18a #23 and Ago18b #15 represent nucleotides not shown.

We first tested the platform for targeted mutagenesis on two closely related but polymorphic Argonaute (*Ago*) genes *ZmAgo18a* (GRMZM2G105250) and *ZmAgo18b* (GRMZM2G457370) that were implicated in 24‐nt phasiRNA biogenesis in anthers (Zhai *et al*., 2015). To enhance mutagenesis success in the targeted exon, two closely located target sites in each *Argonaute* gene were selected for gRNA construction. *ZmAgo18a* was specifically targeted by gAgo18a‐1/gAgo18a‐2 in pgRNA‐IM1, and *ZmAgo18b* was specifically targeted by gAgo18b‐1/gAgo18b‐2 in pgRNA‐IM2 (Figure 3a). A third plasmid targeting both copies of Ago18 simultaneously was constructed by subcloning of the gAgo18a‐1/gAgo18a‐2 cassette from pgRNA‐IM1 into pgRNA‐IM2, which already contained gAgo18b‐1/gAgo18b‐2. The three gRNA constructs were subsequently moved to pGW‐Cas9 through Gateway recombination. For simplicity, the three constructs are referred to as gAgo18a, gAgo18b and gAgo18a/b.

**Figure 4:**
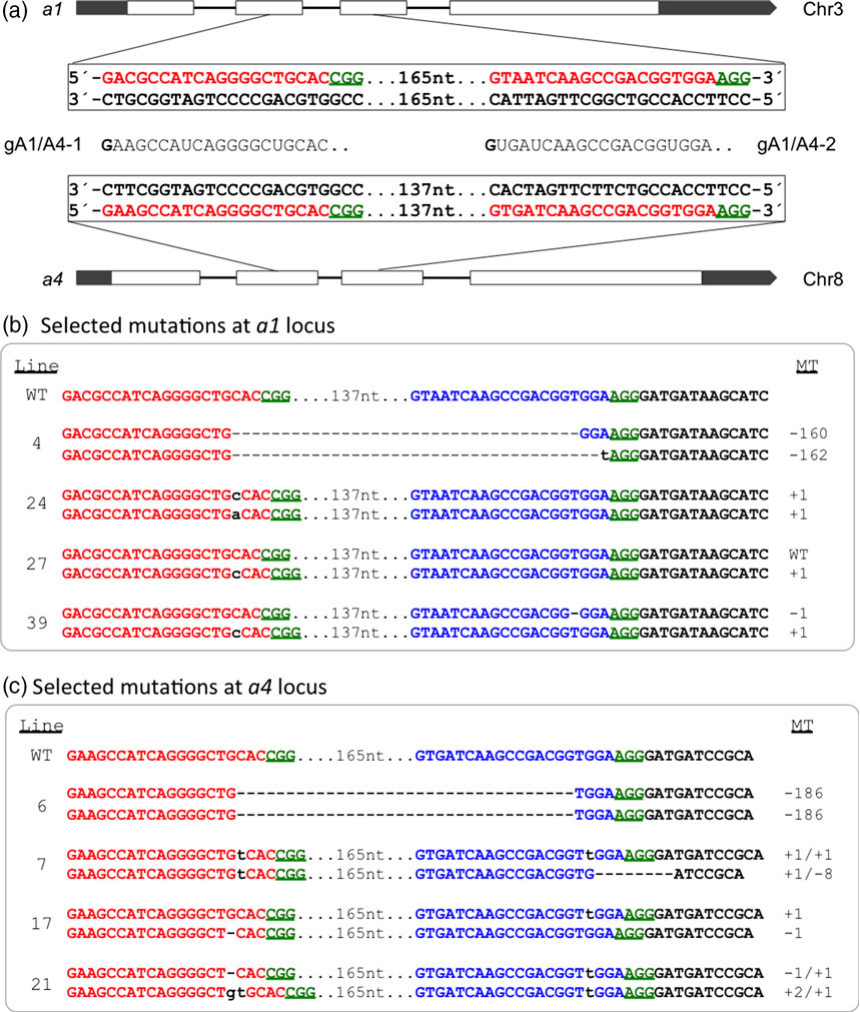
Cas9/gRNA‐induced mutations at the *a1* (chromosome 3) and *a4* (chromosome 8) target sites. (a) Gene structures of *a1* and *a4* loci with gRNAs designed for DSBs in exons (blank bars). Nucleotides in red represent target sites, and green underlined nucleotides indicate PAM sequences for the gRNAs. gRNAs, gA1/A4‐1 and gA1/A4‐2 are between *a1* and *a4* gene boxes. 165 nt and 137 nt represent the numbers of nucleotides between the two target sites in each gene. (b) and (c) Sequences from selected T_0_ plants containing the site‐specific mutations. MT, mutant types; the nucleotide changes (dashes for deletion and lowercase letter in blue for insertion) are also indicated to the right side of each sequence, suffixed with a letter, if needed, to distinguish different alleles. Line, mutant line.

We also constructed gRNAs that targeted the *dihydroflavonol 4‐reductase* or anthocyanin biosynthesis gene *a1* (*anthocyaninless 1*) and its homolog *a4*, two duplicated orthologs of the Arabidopsis *Ben1* gene, encoding a dihydroflavonol 4‐reductase that governs the levels of endogenous brassinosteroid hormones (Yuan *et al*., 2007). The predicted protein sequences encoded by maize *a1* (GRMZM2G026930) and *a4* (GRMZM2G013726) share 88.3% similarity. The two gRNAs (gA1/A4‐1 and gA1/A4‐2) were designed to target conserved sites with a perfect match to *a4* but a mismatch to *a1* in position 3 at the 5′ end of each guide RNA (Figure 4a). Polymorphisms in the target regions between the two genes allowed specific amplification of the relevant regions for genotyping of individual genes.

The constructs were transferred to *Agrobacterium* strain EHA101 and used to infect immature Hi‐II maize embryos. Bialaphos‐resistant callus lines were identified, putative mutants were molecularly analysed, and mutation‐positive callus lines were transferred to regeneration medium, yielding multiple plantlets per line. Plants were brought to maturity and self‐pollinated or reciprocally crossed to a wild‐type line for seeds. During plant growth in the greenhouse, successive leaf samples were taken from two randomly selected plants of each line, and these were combined and used for genomic DNA (gDNA) extraction. PCR amplicons of *ZmAgo18a*,* ZmAgo18b*,* a1* and *a4* targeted regions were analysed by the T7E1 assay and sequenced.

Given the complexity of possible results, from zero to two target sites of one gene (or locus) and 0–4 target sites of two genes (or loci), we have adopted the following terminology to describe the allelic status of transformants. Monoallelic mutants, designated MA, have a mutation in one allele of the target gene (or locus) regardless of the site of the allele and an unmutated second allele. Diallelic mutants, designated DA, have mutations in both homologous copies of the target gene (or locus). For lines with two paralogous genes (or loci) targeted, we simply present the mutations in a combination of MA or DA for each locus. Interpretation of mono‐ and diallelic cases sometimes can be difficult in the T_0_ generation; self‐crossing or outcrossing to another line and the loss of Cas9/gRNA by segregation simplify the interpretation of the T_1_ DNA sequencing results and are used routinely for verification of the interpretation of T_0_ sequences at the target sites.

The two single‐gene targeting constructs gAgo18a and gAgo18b achieved similar transformation and mutagenesis frequencies (Table 1). In the T7E1 assay on selected bialaphos‐resistant calli 74% (17 of 23) of gAgo18a lines and 70% (16 of 23) of gAgo18b lines were scored as mutated. Of the 17 mutated gAgo18a lines, 12 were MA and 5 were DA mutants. Similarly, nine MA mutants and seven DA mutants were identified among the 16 gAgo18b lines. The T7E1‐positive PCR amplicons were subjected to Sanger sequencing and found to contain various combinations of mutations as illustrated for representative lines in Figure 3b, e. A majority of mutant plants tested (e.g. 7/10 of *ZmAgo18b*) contained mutations identical to those detected in the progenitor callus (Table 1). This result suggests that for most events, mutations occurred in a single cell from which each bialaphos‐resistant callus was derived, rather than occurring sporadically during the subsequent callus growth.

**Table 1:** Summary of CRISPR mutagenesis frequencies on four genes in maize Hi‐II genotype

gRNA	Target gene	# bar+ callus line analysed	# Mutation+ callus line	% Mutation frequency	# Monoallelic mutant	# Diallelic mutant	# Mutation+ line regenerated
gAGO18a	*ZmAgo18a*	23	17	74	12	5	17
gAGO18b	*ZmAgo18b*	23	16	70	9	7	16
gAGO18a/b	*ZmAgo18a*	26	3	12	1	2	22
	*ZmAgo18b*		4	15	3	1	
	*ZmAgo18a*&*18b*		15	58	11 (*18a*), 10 (*18b*)	4 (*18a*),5 (*18b*)	
gA1/A4	*a1*	47	7	15	1	6	35
	*a4*		23	49	1	20	
	*a1* & *a4*		7	15	0 (*a1*), 0 (*a4*)	7 (*a1*), 7 (*a4*)	

As noted above in vector design, two gRNAs were used to mutate each *ZmAgo18* gene; the two gRNA targets were separated by 10 nucleotides (nt) in *ZmAgo18a* and by 76 nt for *ZmAgo18b* (Figure 3a). Interestingly, mutations were detected at both target sites for *ZmAgo18a* (Figure 3b, c). However, only one of two gRNAs for *ZmAgo18b* was effective, because all mutations detected in *ZmAgo18b* were located in the target site 1 (Figure 3d, e). It is unknown whether the distance between the two gRNAs or an aspect of gAgo18b‐2 structure contributed to the lack of mutations from this gRNA.

Table 1 summarizes two duplex targeting experiments for *ZmAgo18a* and *ZmAgo18b*, as well as parallel experiments for *a1* and *a4*. In the gAgo18a/b experiment, a total of 22 mutated lines were identified from 26 bialaphos‐resistant callus lines, an 85% frequency of mutagenesis. Among the 22 lines, 12% contained only mutations in *ZmAgo18a*, representing one MA and two DA mutants. Fifteen per cent involved only *ZmAgo18b*, with three MA mutants and one DA mutant. Most mutated lines (58%) had mutations in both *ZmAgo18* genes: 11 MA and 4 DA for *ZmAgo18a* and 10 MA and 5 DA for *ZmAgo18b*.

The design of the *a1*/*a4* duplex targeting experiment was different, with each gRNA targeting sites conserved between the two genes. We achieved a 79% mutagenesis frequency with 37 callus mutants confirmed from 47 bialaphos‐resistant callus lines. Thirty‐five of the 37 callus lines were regenerated and produced plants (Table 1). Similar to gAgo18a/b, gA1/A4 produced three groups of callus‐mutant lines: *a1* single, *a4* single and *a1* and *a4* double mutants. Fifteen per cent of mutations involved only *a1*; 49% were in *a4* only, three times higher than that in *a1*. The lower mutation efficiency in *a1* is likely attributable to the 1‐bp mismatch between the target sequence of *a1* and each of two gRNAs (Figure 4a). Nine lines (15%) involved both the *a1* and *a4* genes. Various combinations of MA and DA mutations were observed. Figure 4b, c shows the mutations identified for selected lines.

### Inheritance of Cas9/gRNA‐mediated mutations

Individual T_0_ plants from selected mutant lines were self‐pollinated or cross‐pollinated with the maize inbred line B73. Once mutated, target sites should no longer be recognized by gRNA and therefore not subject to further rounds of mutagenesis. To avoid the complications of continuing action by the Cas9/gRNA transgene in B73 alleles introduced by cross‐pollination, we chose populations derived from selfing of T_0_ lines carrying homogeneous or heterogeneous DA mutations for transmission analysis. For genotyping T_1_ plants, 20 seeds from independent transgenic events were germinated and grown in the greenhouse. Genomic DNA was extracted and PCR amplicons from the targeted region were examined using the T7E1 assay and, for a subset of samples, Sanger sequencing of amplicons was performed.

Inheritance of the mutated *ZmAgo18a* and *ZmAgo18b* alleles was analysed in independent lines. Table 2 shows inheritance results for four selected lines, two *ZmAgo18a* mutants (Ago18a #2 and #15) and two *ZmAgo18b* mutants (Ago18b #19 and #20). For all four lines, mutations in the T_0_ plants were transmitted to the T_1_ generation. Ago18a #2 was a DA mutant: one mutated allele had a 51‐bp deletion and the second allele had a 35‐bp deletion. Ago18a#15 was a homogenous DA mutant with a 5‐bp deletion (Figure 3b); thus, there was no segregation for mutations among the progeny (Table 2). Ago18b #19 was DA of 1‐bp deletion and 1‐bp insertion, while Ago18b #20 was DA for heterogeneous 2‐ and 4‐bp deletions (Figure 3e). For the inheritance of Cas9/gRNA, *Cas9* was analysed using a PCR approach with gene‐specific primers. If T_0_ plants had a single transgene locus, *Cas9* would be expected to segregate 3 : 1 in the T_1_ progeny from the self‐pollination; this Mendelian expectation was confirmed in Ago18a #2, Ago18b #19 and #20. However, all 20 seedlings from Ago18a #15 carried the Cas9/gRNA transgene (Table 2). This type of non‐Mendelian segregation could result from transgenic lines with multiple transgene copies integrated on different chromosomes and could also occur if a sterility mutation eliminates noncarrier pollen, that is via an unselected mutation from transformation or tissue culture in repulsion to the Cas9/gRNA transgene.

**Table 2:** Transmission of Cas9/gRNA‐induced mutations in T1 progeny

Lines^a^	# analysed	*Cas9* positive			*Cas9* negative			*Cas9* pos vs *Cas9* neg	*P*‐value^b^
		aa	ab	bb	aa	ab	bb		
AGO18a #2	20	6	7	5	1	1	0	18 : 2	0.121
AGO18a #15	20	20	0	0	0	0	0	20 : 0	0.010
AGO18b #19	18	1	12	3	0	0	2	16 : 2	0.174
AGO18b #20	16	3	5	4	2	0	2	12 : 4	1.000

aa & bb, homozygous a and b; ab, heterozygous mutant ab.

a All mutant lines were self‐pollinated; expected segregation ratio is 3 : 1 (*Cas9* positive: *Cas9* negative).

b Chi‐square probability with one degree of freedom.

### Inherited Cas9 and gRNA expression induces new mutations in progeny plants

**Figure 5:**
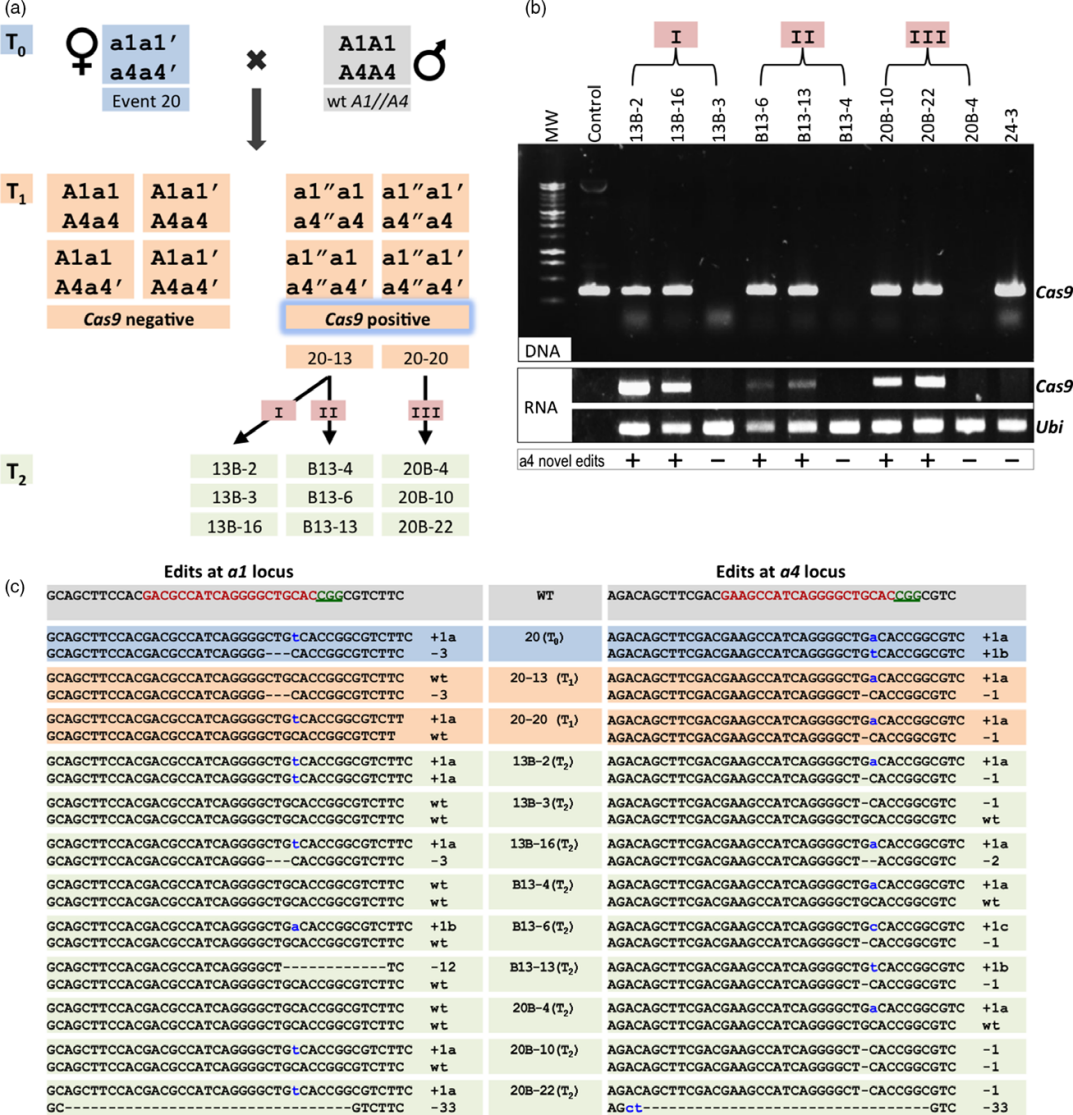
Characterization of sexually heritable new alleles after targeted mutagenesis by Cas9/gRNA in maize cells. (a) Schematic diagram showing the inheritance and segregation of original edited alleles as well as the generation of new mutations from *a1//a4* CRISPR event 20. The T_0_ has DA mutations for both *a1* and *a4*. The T_1_ and T_2_ progeny were derived by crossing mutants to recipient lines with wild‐type *a1* and *a4* loci. The wild‐type allele is represented as ‘A’, the T_0_ mutated alleles as ‘a’ and ‘a′’. ‘a″’ indicates alleles that potentially contain novel mutations. For the development of the T_2_ generation, T_1_ plant 20‐13 was used as a female (cross I), or male (cross II), and T_1_ plant 20‐20 was used as a female (cross III). (b) The top panel shows the presence of *Cas9* in genomic DNA in the T_2_ progeny plants of event 20 as assayed by PCR. The control lane represents a plasmid‐positive control with cloned *Cas9*. The bottom panel shows *Cas9* transcript levels by RT‐PCR. The control lane represents ‐RT (negative control), and *ubiquitin 1* gene expression (Ubi) serves as the positive control. ‘+’ stands for the presence of novel mutation in T_2_, and ‘−’ stands for its absence. (c) Sequence information at the *a1* and *a4* targeted loci for the T_0_, T_1_ and T_2_ plants from event 20. Nucleotides in red represent target sites and green underlined nucleotides indicate PAM sequences for the gRNAs. The nucleotide variations (dashes for deletion and lowercase letter in blue for insertion) are marked on the right side of each sequence with a number, suffixed with a letter, if needed, to distinguish different alleles. Line names are listed in the middle column.

To investigate whether the Cas9/gRNA transgene cassette remains active and can induce mutations after the carrier plant is crossed to a wild‐type inbred line, we chose one *a1/a4* mutation‐positive line (#20) for analysis. This line was chosen because it has DA mutations in both the *a1* and *a4* genes (Figure 5a, c, blue T_0_). Female flowers of T_0_ plants were crossed by using pollen from B73 carrying wild‐type *a1* and *a4* alleles. As shown in Figure 5a, the T_1_ progeny population can be divided into *Cas9*‐positive (transgenic lines) and *Cas9*‐negative (transgene null) segregants. Two of eight T_1_ plants screened (25%) carried the Cas9/gRNA transgene. DNA sequencing analysis of the two *Cas9*‐positive individuals indicated that both had novel mutations in *a4*, but not in *a1* (Table 3). The novel mutations were verified from three independent leaves of the same plant, indicating that the mutations did not reflect mosaicism (Figure 5a, c). This observation suggests that the novel mutations in the wild‐type B73 allele occurred early in development, perhaps in the zygote.

**Table 3:** Novel mutations in Cas9/gRNA‐positive progenies

Outcross with wild‐type *a1//a4*		Total # screened	*Cas9* pos	Novel mutations (%)	
				*a1*	*a4*
T_1_ generation					
I	Event 20 as female	8	2	0 (0%)	2 (100%)
T_2_ generation					
I	Line 20–13 as female	17	9	7 (78%)	9 (100%)
II	Line 20–13 as male	19	12	10 (83%)	12 (100%)
III	Line 20–20 as female	24	12	7 (58%)	12 (100%)
T_2_ total		60	33	24 (73%)	33 (100%)

To further confirm the heritable activity of the Cas9/gRNA transgene, *Cas9*‐positive plants from the two T_1_ lines, designated as 20‐13 and 20‐20, were further outcrossed to B73 wild‐type plants to generate T_2_ progeny. Plant 20‐13 was crossed with B73 either as female (Figure 5a, cross I) or as male (Figure 5a, cross II) to assess the efficiency of novel mutation generation and of Cas9 activity in reciprocal crosses. Plant 20‐20 was the ear parent in crosses with B73 (Figure 5a, cross III).

A total of 60 T_2_ generation plants from lines 20‐13 and 20‐20 were screened for the presence of *Cas9*, and novel mutations were detected at the *a1* and *a4* loci, which revealed continued mutagenesis at high frequencies (Table 3). As expected, segregation for the *Cas9* transgene was approximately 1 : 1, with 33 of the 60 plants carrying *Cas9*. All *Cas9*‐positive plants carried novel mutations in the *a4* gene, but only a subset (58%–83%) also carried mutations in *a1*. Preferential mutation of *a4* was also observed in the T_1_ generation (Table 3), an observation consistent with the T_0_ generation analysis (Table 1).

RT‐PCR‐based expression analysis of *Cas9* in the T_2_ progeny demonstrated a positive correlation between the presence of *Cas9* mRNA and the occurrence of novel mutations. As can be seen in Figure 5b, c, all T_2_ lines that showed continued mutagenesis contained an actively transcribed *Cas9*, whereas no further mutations in *a1* or *a4* genes were detected in lines lacking the *Cas9* transgene (13B‐3, B13‐4, 20B‐4). Notably, continued mutating was not detected in one line (24‐3) that contained a *Cas9* transgene. However, no *Cas9* expression was detectable in this plant (Figure 5b, Figure S1), indicating that inheritance of the *Cas9* transgene is not sufficient and that *Cas9* expression is also required for mutagenesis.

### Co‐infection of two *Agrobacterium* strains harbouring different Cas9/gRNA constructs produces respective mutations

Given the high efficiency of our Cas9/gRNA system in the initial transformations, we explored the feasibility of mutating two genes (or two groups of genes) in one infection procedure by mixing *Agrobacterium* strains harbouring different gRNA constructs for co‐transformation. The motivation for this was to reduce the number of embryos required and the cost of plant transformation while still producing an adequate number of mutants for each gene or group of genes. This represents an alternative strategy for multiplex targeting which can also be achieved with multiple gRNAs in one construct. As a proof‐of‐concept experiment, two *Agrobacterium* EHA101 strains, one containing Cas9/gAgo18a and another Cas9/gAgo18b, were mixed to obtain equal bacterial cell density. The mixed bacterial culture was then used to infect a similar number of maize B104 immature embryos as is usually used for a single *Agrobacterium*‐mediated transformation.

A total of twenty‐two independent bialaphos‐resistant calli were identified. These lines were first screened for the presence of Cas9/gRNA transgenes using PCR. Twenty‐one of twenty‐two lines (95%) were positive for the Cas9/gRNA transgenes. Of these twenty‐one calli, nine lines (43%) were positive only for the gAgo18a transgene, nine lines (43%) were positive only for gAgo18b and three lines (14%) were positive for both gAgo18a and gAgo18b transgenes (Table 4). The results indicate that one *Agrobacterium* infection experiment can produce two major groups of transgenic callus lines with a small portion of double transformation.

**Table 4:** Summary of transformation and mutagenesis frequencies in co‐infection experiment

Event ID	Transgene (callus)		Mutant (callus)		Mutant (plant)	
	gAgo18a	gAgo18b	*Ago18a*	*Ago18b*	*Ago18a*	*Ago18b*
1	+	−	+	−	+	−
2	−	+	−	−	−	−
3	+	−	−	−	−	−
4	+	+	−	−	−	+
5	−	+	−	−	−	+
6	+	+	+	−	+	−
7	−	+	−	−	−	−
8	+	−	−	−	+	−
9	+	−	−	−	+	−
10	+	+	+	+	+	−
11	−	+	−	−	−	−
12	−	+	−	+	−	+
13	−	+	−	+	−	−
14	−	+	−	−	−	+
15	+	−	+	−	+	−
16	+	−	−	−	+	−
17	+	−	−	−	+	−
18	+	−	+	−	+	−
19	+	−	+	−	+	−
20	−	+	−	−	−	+
21	−	+	−	−	−	−
Total	12	12	6	3	10	5
Efficiency	Transformation		Mutagenesis		Mutagenesis	
	57%	57%	29%	14%	48%	24%

Transformed calli were further analysed for mutations in the targeted genes using both the T7E1 assay and Sanger sequencing. Among those lines carrying Cas9/gRNA transgenes, five were positive for mutations of *ZmAgo18a*, two for *ZmAgo18b* and one for both genes. On the other hand, neither callus line carrying both transgenes (lines 4 and 6) produced mutations in both genes. This discrepancy could be attributed to issues related to false negatives in PCR analysis or incomplete sampling of representative callus cells.

Multiple plantlets (3–5) per callus line were regenerated from the 21 transgenic lines. Two to three plantlets derived from the same transformed callus line were randomly selected and individually analysed for mutations in the target genes. As can be seen in Table 4, seven (47%) of 15 lines that were mutation positive in the callus tissue for *ZmAgo18a* and *18b* were also mutation positive in plantlets. These include five lines positive to *ZmAgo18a* (lines 1, 6, 15, 18 and 19) and one line positive for *Ago18b* (line 12); no line was positive for both genes. One line (line 13) was positive for *Ago18b* in callus assay, but the mutation was not detected in plants. On the other hand, eight lines (lines 4, 5, 8, 9, 14, 16, 17 and 20) tested negative for mutations in the callus, but the plants were found to contain mutations in their respective genes. These results indicate that the initial callus assays are reasonable predictors of plant genotype; however, assay improvements may be warranted to increase the accuracy or analysis of multiple regions of calli to determine whether there is unexpected chimerism.

These results from this co‐infection experiment indicate that mixing two *Agrobacterium* strains generates mutation frequencies in individual target genes of respective Cas9/gRNA similar to single strain infections. This is consistent with earlier findings showing that when two *Agrobacterium* strains were used for co‐infection, the majority of transgenic plants obtained carry only one type of T‐DNA in their genome (De Buck *et al*., 2009; De Neve *et al*., 1997). Hence, the co‐infection approach can be exploited to effectively induce mutations in individual target genes (or groups of conserved genes) and produce mutant plants, thus reducing transformation costs and increasing throughput.

## Discussion

We report a high‐efficiency CRISPR platform, ISU Maize CRISPR, consisting of Cas9/gRNA utilized with *Agrobacterium*‐mediated transformation for targeted mutagenesis of maize in a 7‐month process. Efficacy was tested with four maize genes: combining all results, 60% of transgenic callus lines contained site‐specific mutations that persisted in regenerated plants and were heritable in the T_1_ generations. The Cas9/gRNA transgene, when mobilized to the B73 inbred via genetic crosses, could induce new heritable mutations in wild‐type alleles. Finally, co‐infection of two *Agrobacterium* strains harbouring distinct Cas/gRNA constructs generated mutations in the respective target genes with frequencies similar to those observed in single transformations. We expect that this highly efficient and cost‐effective CRISPR platform will become an enabling genomic tool for the public research community.

gRNA‐directed Cas9, like other types of engineered nucleases (e.g. meganucleases, ZFNs and TALENs), has been engineered to induce site‐specific DSBs in a host genome, wherein NHEJ is the predominant repair mechanism. The propensity of NHEJ for introducing small indels leads to NHEJ‐based genomic mutagenesis as the major application in genome editing (Sander and Joung, 2014). In contrast to the endonucleases first deployed, Cas9/gRNA transgenes are simpler to construct and are more efficient mutagens. Our experience with the same maize transformation platform, but a different gene target than either *Ago18* or *a1/a4*, indicates that mutagenesis frequency by TALENs is about 10% in Hi‐II and 3.7% in B104 (Char *et al*., 2015), which is at least sixfold lower than reported here for Cas9/gRNA‐mediated events. On the other hand, the reported mutagenesis efficiencies of different species or even different maize CRISPR platforms differ significantly. For example, relatively low mutagenesis frequency (5.6%) was reported in wheat, a cereal crop with a large and complex genome (Wang *et al*., 2014). In contrast, much higher mutagenesis rates (up to 100%) for rice have been reported by a number of laboratories (Zhou *et al*., 2014; Zhang *et al*., 2014; Xie *et al*., 2015; Ma *et al*., 2015). In dicotyledonous tomato and soybean, CRISPR systems produced more than 50% mutated T_0_ plants (Brooks *et al*., 2014; Li *et al*., 2015). Among the reported maize CRISPR systems, frequencies of 2%–100% were reported in the T_0_ Cas9‐positive plants (Feng *et al*., 2016; Liang *et al*., 2014; Svitashev *et al*., 2015; Xing *et al*., 2014; Zhu *et al*., 2016). These differences reflect a variety of factors that influence the frequency of NHEJ‐mediated mutagenesis. The main factors impacting this efficiency are different promoters that direct the spatiotemporal expression of *Cas9* and gRNAs, methods of Cas9/gRNA delivery that result in different transgene copy numbers and thus variation in the host cell abundance of the Cas9/gRNA ribonucleoprotein complex and, finally, the specific target gene sequence and chromosomal location, which affects the accessibility the target gene to Cas9/gRNA ribonucleoprotein to cause DSBs (Wu *et al*., 2014; Horlbeck *et al*., 2016). Nevertheless, our Cas9/gRNA system showed similarly high efficiency with all four genes tested and with an additional 27 constructs targeting 30 maize genes (unpublished data).

The current necessity for plant transformation makes maize genome editing an expensive, laborious and time‐consuming process. We adapted three strategies to improve efficiency in terms of the cost of consumables and labour. First, we designed and constructed two gRNAs targeting each gene to increase (presumably double) the success rate or improve the possibility that at least one gRNA will be active for mutagenesis. Our experience in CRISPR‐mediated mutagenesis in plants indicates that not every gRNA constructed is active or highly active *in planta* to induce DSBs and subsequently targeted mutagenesis. For example, in this work, only one (gAgo18b‐1) of the two gRNAs constructed for targeting *ZmAgo18b* was mutagenic. Until a simple and rapid assay to identify the most effective gRNA candidate is available prior to maize transformation, the approach described here of using two gRNAs for one gene would improve the odds of achieving targeted mutagenesis. Another important utility of the 2‐gRNAs‐for‐1‐gene approach is to enable large deletion mutation in the targeted gene. Different vector systems and cloning approaches can be used to enhance the efficiency of the multiple gRNA construction process. For example, the Golden Gate assembly technology can be adapted to make multiple gRNA expression units in one reaction and in either the gRNA intermediate vector or directly in the destination Cas9 vector (Engler *et al*., 2008).

Second, we incorporated a genotyping procedure to screen bialaphos‐resistant callus lines and retain only mutation‐positive lines for regeneration. The callus screening is an important step for resource conservation especially when performing the transformation of inbred B104. Compared to the Hi‐II transformation, regeneration of transgenic B104 callus is technically demanding, time‐consuming and requires extended use of growth chambers. Therefore, the early identification of mutation‐positive callus lines will maximize the number of CRISPR plants for each mutagenesis project. In our work, the majority of sequencing chromatograms showed two predominant peaks starting at the expected mutation sites, indicating uniform genotypes within calli, including some lines containing homogenous or heterogeneous DA mutations. Over 70% of edit‐positive callus lines remained positive for mutations in the regenerated plants. Third, we tested the feasibility of combining two independent mutageneses through co‐incubation of two *Agrobacterium* strains with the standard number of starting embryos and showed that each independent mutagenesis occurred at frequencies similar to those for individual infections. The possibility of combining more than two different Cas9/gRNA‐containing *Agrobacterium* strains needs to be further tested for efficacy.

In this study, we analysed the progeny between integrated Cas9/gRNA in T_0_ and T_1_ plants crossed with B73 for heritable activity of the mutagenesis reagents. In both populations, novel mutations at specific genomic sites were detected at frequencies ranging from 0% to 100%. This continuing action of Cas9/gRNA is in agreement with previous reports of new mutations (Brooks *et al*., 2014; Svitashev *et al*., 2015; Wang *et al*., 2014; Zhang *et al*., 2014). Heritable Cas9/gRNA action points to the prospect of performing intergenotype targeted mutagenesis for some applications. For example, transgenic B104 carrying a specific Cas9/gRNAs might be crossed with transformation‐recalcitrant maize genotypes to generate desired mutagenesis in specific alleles in the Cas9/gRNA recipient maize. This approach could also facilitate other genetic procedures, such as introgression of a recessive trait generated by Cas9/gRNA into specific germplasm, or performance of double‐mutant analyses. Furthermore, once a *Cas9‐*expressing plant exists, transient introduction of gRNAs by any means could permit gene modification for any chosen target without the necessity of generating a new maize transformant.

With our efficient and robust CRISPR platform, ISU Maize CRISPR, transgenic maize plantlets with gene‐specific mutations can be generated as early as 16 weeks from the day of construct delivery after which seed can be produced in an additional 3–4 months. Transgenic plants with mutations are typically pollinated with wild‐type donor pollen to produce segregants that have gene‐specific mutations, but are free of the Cas9/gRNA transgene. These null‐segregant mutant seeds containing no foreign DNA sequences can then be used for further fundamental and applied research, with minimal or no regulatory and containment requirements. We expect that this *Agrobacterium*‐delivered ISU Maize CRISPR system will empower the public research community and accelerate the exploration of both gene function and trait improvement.

## Experimental procedures

### Plasmids and bacterial strains

Molecular cloning and the construction of maize transformation plasmids were performed as previously described (Ausubel *et al*., 1993). The transformation vector was based on pMCG1005 (kindly provided by Dr. Vicki Chandler), a binary vector for *Agrobacterium*‐mediated maize transformation containing four copies of an enhanced CaMV 35S promoter driving *bar* gene expression for bialaphos resistance. pMCG1005 also contains a cassette of the maize *ubiquitin 1* gene promoter (Christensen and Quail, 1996) coupled with the first intron of maize alcohol dehydrogenase (*Adh1*) gene (Callis *et al*., 1987) and the terminator of octopine synthase gene of *Agrobacterium tumefaciens* (Koncz *et al*., 1983). This vector was modified by replacing the first intron of the rice waxy gene with a linker sequence to facilitate the cloning of *Cas9*. The rice codon‐optimized *Cas9* was as described (Jiang *et al*., 2013). Additionally, the Gateway recombination cassette of attR1‐ccdB‐attR2 was cloned into pMCG1005, resulting in the destination vector, pGW‐Cas9. Sequence information is available upon request.

For the construction of guide RNA genes, the intermediate vectors pENTR‐gRNA1 and pENTR‐gRNA2 that each can express two gRNAs were used (Zhou *et al*., 2014). Briefly, in each gRNA vector, a cloning site with 2×*Btg*ZI downstream of one rice U6 promoter and another cloning site with 2×*Bsa*I downstream of the second rice U6 promoter were used for sequential insertions of two gRNA spacer sequences (Zhou *et al*., 2014). To construct the gRNA gene targeting a specific genomic locus, two complementary oligonucleotides (21–25 nt) were annealed to produce a double‐stranded DNA oligonucleotide (dsOligo). For the *Btg*ZI cloning site, the sense strand contains a 5′ 4‐nt overhang of TGTT and the antisense strand contains a AAAC 5′ overhang. For the *Bsa*I cloning site, the double‐stranded oligonucleotides were designed with a GTGT 5′ overhang on the sense strand and AAAC 5′ overhang on the antisense strand. All oligonucleotides were synthesized at Integrated DNA Technology (Coralville, IA). The first dsOligo was inserted into *Btg*ZI restriction site and the second dsOligo was sequentially inserted at the *Bsa*I restriction site followed by sequencing to confirm the accuracy of construction. To construct a gRNA cassette expressing four guide RNA genes, the gRNA cassette from pgRNA‐IM1 was cut out using *Hin*dIII and subcloned into pgRNA‐IM2 that was already constructed to contain two gRNA sequences. The gRNA cassettes were finally mobilized to pGW‐Cas9 by using the Gateway LR Clonase (Thermo Fisher Scientific, Waltham, MA).

*Escherichia coli* strain XL1‐Blue was used for molecular cloning of Cas9/gRNA constructs and *Agrobacterium tumefaciens* strain EHA101 for maize transformation. *Escherichia coli* cells were grown in Luria–Bertani (LB) medium at 37 °C with a standard culture technique (Ausubel *et al*., 1993), and *Agrobacterium* was grown at 28 °C in YEP medium (yeast extract 5 g/L, peptone 10 g/L, NaCl_2_ 5 g/L) with appropriate antibiotics.

### Maize tissue culture and transformation

Maize (*Zea mays*) hybrid genotype Hi‐II and inbred line B104 were used. *Agrobacterium*‐mediated transformation of the immature embryos of Hi‐II and B104 maize genotypes was performed at the Iowa State University Plant Transformation Facility as described (Frame *et al*., 2002, 2006). The plants were grown in greenhouses with controlled temperatures of 26 °C/22 °C and a photoperiod of 16 h/8 h (day/night).

### Genotyping maize callus lines and plants

Genomic DNA from maize calli and leaves of transgenic plants was extracted using the cetyltrimethyl ammonium bromide (CTAB) method (Murray and Thompson, 1980). Genomic DNA was used for PCR amplification of relevant regions with specific primers flanking the target sites (Table S1). PCR reaction conditions were optimized for each primer pair and are available upon request. PCR amplicons were assessed for mutations using the T7 endonuclease I (T7E1) assay and Sanger sequencing. PCR amplicons obtained from the transgenic tissues were mixed with the respective amplicon derived from wild type, denatured (95 °C for 5 min) and reannealed (ramp down to 25 °C at 5 °C/min), then subjected to T7E1 digestion and agarose gel electrophoresis as previously described (Char *et al*., 2015). The amplicons derived from the T7E1‐positive samples were treated with ExoSAP‐IT (Affymetrix, Santa Clara, CA) and subsequently evaluated by the Sanger sequencing method by the Iowa State University DNA facility (http://www.dna.iastate.edu/). The sequencing chromatograms were carefully examined for exact patterns that might indicate monoallelic or diallelic mutations.

### Expression analysis in progeny plants by RT‐PCR

The expression of Cas9 mRNA in progeny plants was evaluated by RT‐PCR on an Eppendorf Mastercycler (Eppendorf, Hamburg, Germany). Total RNA was isolated from one‐week‐old seedlings using RNeasy Plant Mini Kit (Qiagen, Valencia, CA) as per the manufacturer's instructions. The concentration and purity of the isolated RNA was confirmed by NanoDrop ND‐1000 Spectrophotometer. One microgram of RNA was subjected to DNase treatment with Promega RQ1 RNase‐free DNase I (Promega Corporation, Madison, WI) followed by reverse transcription mediated by SuperScript^™^ III Reverse Transcriptase (Thermo Fisher Scientific, Waltham, MA) following the manufacturer's protocol. The cDNA was amplified using OsCas9 primers (Table S1) for 26 cycles, and the products were separated on agarose gels, visualized by SYBR Safe DNA gel stain (Thermo Fisher Scientific) and photographed. The expression of maize *ubiquitin* cDNA was also determined in the same sample set, as an endogenous positive control.

## Conflict of interest

The authors declare no conflict of interest.

## Supporting information


**Figure S1** *a1* and *a4* Genotypes of CRISPR line 24‐3.
**Table S1** Primers and sequences used in this study.

## References

[pbi12611-bib-0001] Ausubel, F. , Brent, R. , Kingston, R. , Moore, D. , Seidman, J. and Struhl, K. (1993) Current Protocols in Molecular Biology, 1st ed. New York: Canada John Wiley and Sons.

[pbi12611-bib-0002] Bhaya, D. , Davison, M. and Barrangou, R. (2011) CRISPR‐Cas systems in bacteria and archaea: versatile small RNAs for adaptive defense and regulation. Annu. Rev. Genet. 45, 273–297. 22060043 10.1146/annurev-genet-110410-132430

[pbi12611-bib-0003] Brazelton, V.A. Jr. , Zarecor, S. , Wright, D.A. , Wang, Y. , Liu, J. , Chen, K. , Yang, B. , *et al*. (2015) A quick guide to CRISPR sgRNA design tools. GM Crops Food, 6, 266–276. 26745836 10.1080/21645698.2015.1137690 PMC5033207

[pbi12611-bib-0004] Brooks, C. , Nekrasov, V. , Lippman, Z.B. and Van Eck, J. (2014) Efficient gene editing in tomato in the first generation using the clustered regularly interspaced short palindromic repeats/CRISPR‐associated 9 system. Plant Physiol. 166, 1292–1297. 25225186 10.1104/pp.114.247577 PMC4226363

[pbi12611-bib-0005] Callis, J. , Fromm, M. and Walbot, V. (1987) Introns increase gene expression in cultured maize cells. Genes Dev. 1, 1183–1200. 2828168 10.1101/gad.1.10.1183

[pbi12611-bib-0006] Cermak, T. , Baltes, N.J. , Cegan, R. , Zhang, Y. and Voytas, D.F. (2015) High‐frequency, precise modification of the tomato genome. Genome Biol. 16, 232. 26541286 10.1186/s13059-015-0796-9 PMC4635538

[pbi12611-bib-0007] Char, S.N. , Unger‐Wallace, E. , Frame, B. , Briggs, S.A. , Main, M. , Spalding, M.H. , Vollbrecht, E. , *et al*. (2015) Heritable site‐specific mutagenesis using TALENs in maize. Plant Biotechnol. J. 13, 1002–1010. 25644697 10.1111/pbi.12344

[pbi12611-bib-0008] Christensen, A.H. and Quail, P.H. (1996) Ubiquitin promoter‐based vectors for high‐level expression of selectable and/or screenable marker genes in monocotyledonous plants. Transgenic Res. 5, 213–218. 8673150 10.1007/BF01969712

[pbi12611-bib-0009] De Buck, S. , Podevin, N. , Nolf, J. , Jacobs, A. and Depicker, A. (2009) The T‐DNA integration pattern in Arabidopsis transformants is highly determined by the transformed target cell. Plant J. 60, 134–145. 19508426 10.1111/j.1365-313X.2009.03942.x

[pbi12611-bib-0010] De Neve, M. , De Buck, S. , Jacobs, A. , Van Montagu, M. and Depicker, A. (1997) T‐DNA integration patterns in co‐transformed plant cells suggest that T‐DNA repeats originate from co‐integration of separate T‐DNAs. Plant J. 11, 15–29. 9025300 10.1046/j.1365-313x.1997.11010015.x

[pbi12611-bib-0011] Engler, C. , Kandzia, R. and Marillonnet, S. (2008) A one pot, one step, precision cloning method with high throughput capability. PLoS ONE, 3, e3647. 18985154 10.1371/journal.pone.0003647 PMC2574415

[pbi12611-bib-0012] Feng, Z. , Zhang, B. , Ding, W. , Liu, X. , Yang, D.L. , Wei, P. , Cao, F. , *et al*. (2013) Efficient genome editing in plants using a CRISPR/Cas system. Cell Res. 23, 1229–1232. 23958582 10.1038/cr.2013.114 PMC3790235

[pbi12611-bib-0013] Feng, Z. , Mao, Y. , Xu, N. , Zhang, B. , Wei, P. , Yang, D.L. , Wang, Z. , *et al*. (2014) Multigeneration analysis reveals the inheritance, specificity, and patterns of CRISPR/Cas‐induced gene modifications in Arabidopsis. Proc. Natl Acad. Sci. USA, 111, 4632–4637. 24550464 10.1073/pnas.1400822111 PMC3970504

[pbi12611-bib-0014] Feng, C. , Yuan, J. , Wang, R. , Liu, Y. , Birchler, J.A. and Han, F. (2016) Efficient targeted genome modification in maize using CRISPR/Cas9 system. J. Genet. Genom. 43, 37–43. 10.1016/j.jgg.2015.10.002 26842992

[pbi12611-bib-0015] Frame, B.R. , Shou, H. , Chikwamba, R.K. , Zhang, Z. , Xiang, C. , Fonger, T.M. , Pegg, S.E. , *et al*. (2002) *Agrobacterium tumefaciens*‐mediated transformation of maize embryos using a standard binary vector system. Plant Physiol. 129, 13–22. 12011333 10.1104/pp.000653 PMC1540222

[pbi12611-bib-0016] Frame, B.R. , McMurray, J.M. , Fonger, T.M. , Main, M.L. , Taylor, K.W. , Torney, F.J. , Paz, M.M. , *et al*. (2006) Improved *Agrobacterium*‐mediated transformation of three maize inbred lines using MS salts. Plant Cell Rep. 25, 1024–1034. 16710703 10.1007/s00299-006-0145-2

[pbi12611-bib-0017] Horlbeck, M.A. , Witkowsky, L.B. , Guglielmi, B. , Replogle, J.M. , Gilbert, L.A. , Villalta, J.E. , Torigoe, S.E. , *et al*. (2016) Nucleosomes impede Cas9 access to DNA in vivo and in vitro. Elife, 5, e12677. 26987018 10.7554/eLife.12677 PMC4861601

[pbi12611-bib-0018] Hou, Z. , Zhang, Y. , Propson, N.E. , Howden, S.E. , Chu, L.F. , Sontheimer, E.J. and Thomson, J.A. (2013) Efficient genome engineering in human pluripotent stem cells using Cas9 from *Neisseria meningitidis* . Proc. Natl Acad. Sci. USA, 110, 15644–15649. 23940360 10.1073/pnas.1313587110 PMC3785731

[pbi12611-bib-0019] Jiang, W. , Zhou, H. , Bi, H. , Fromm, M. , Yang, B. and Weeks, D.P. (2013) Demonstration of CRISPR/Cas9/sgRNA‐mediated targeted gene modification in Arabidopsis, tobacco, sorghum and rice. Nucleic Acids Res. 41, e188. 23999092 10.1093/nar/gkt780 PMC3814374

[pbi12611-bib-0020] Jiang, W. , Yang, B. and Weeks, D.P. (2014) Efficient CRISPR/Cas9‐mediated gene editing in *Arabidopsis thaliana* and inheritance of modified genes in T2 and T3 generations. PLoS ONE, 9, e99225. 24918588 10.1371/journal.pone.0099225 PMC4053344

[pbi12611-bib-0021] Koncz, C. , De Greve, H. , Andre, D. , Deboeck, F. , Van Montagu, M. and Schell, J. (1983) The opine synthase genes carried by Ti plasmids contain all signals necessary for expression in plants. EMBO J. 2, 1597–1603. 11892818 10.1002/j.1460-2075.1983.tb01630.x PMC555329

[pbi12611-bib-0022] Lawrenson, T. , Shorinola, O. , Stacey, N. , Li, C. , Ostergaard, L. , Patron, N. , Uauy, C. , *et al*. (2015) Induction of targeted, heritable mutations in barley and *Brassica oleracea* using RNA‐guided Cas9 nuclease. Genome Biol. 16, 258. 26616834 10.1186/s13059-015-0826-7 PMC4663725

[pbi12611-bib-0023] Li, Z. , Liu, Z.B. , Xing, A. , Moon, B.P. , Koellhoffer, J.P. , Huang, L. , Ward, R.T. , *et al*. (2015) Cas9‐guide RNA directed genome editing in soybean. Plant Physiol. 169, 960–970. 26294043 10.1104/pp.15.00783 PMC4587461

[pbi12611-bib-0024] Liang, Z. , Zhang, K. , Chen, K. and Gao, C. (2014) Targeted mutagenesis in *Zea mays* using TALENs and the CRISPR/Cas system. J. Genet. Genom. 41, 63–68. 10.1016/j.jgg.2013.12.001 24576457

[pbi12611-bib-0025] Ma, X. , Zhang, Q. , Zhu, Q. , Liu, W. , Chen, Y. , Qiu, R. , Wang, B. , *et al*. (2015) A robust CRISPR/Cas9 system for convenient, high‐efficiency multiplex genome editing in monocot and dicot Plants. Mol. Plant. 8, 1274–1284. 25917172 10.1016/j.molp.2015.04.007

[pbi12611-bib-0026] Murray, M.G. and Thompson, W.F. (1980) Rapid isolation of high molecular weight plant DNA. Nucleic Acids Res. 8, 4321–4325. 7433111 10.1093/nar/8.19.4321 PMC324241

[pbi12611-bib-0027] Sander, J.D. and Joung, J.K. (2014) CRISPR‐Cas systems for editing, regulating and targeting genomes. Nat. Biotechnol. 32, 347–355. 24584096 10.1038/nbt.2842 PMC4022601

[pbi12611-bib-0028] Schnable, P.S. , Ware, D. , Fulton, R.S. , Stein, J.C. , Wei, F. , Pasternak, S. , Liang, C. , *et al*. (2009) The B73 maize genome: complexity, diversity, and dynamics. Science, 326, 1112–1115. 19965430 10.1126/science.1178534

[pbi12611-bib-0029] Shukla, V.K. , Doyon, Y. , Miller, J.C. , DeKelver, R.C. , Moehle, E.A. , Worden, S.E. , Mitchell, J.C. , *et al*. (2009) Precise genome modification in the crop species *Zea mays* using zinc‐finger nucleases. Nature, 459, 437–441. 19404259 10.1038/nature07992

[pbi12611-bib-0031] Svitashev, S. , Young, J.K. , Schwartz, C. , Gao, H. , Falco, S.C. and Cigan, A.M. (2015) Targeted mutagenesis, precise gene editing, and site‐specific gene insertion in maize using Cas9 and guide RNA. Plant Physiol. 169, 931–945. 26269544 10.1104/pp.15.00793 PMC4587463

[pbi12611-bib-0032] Wang, Y. , Cheng, X. , Shan, Q. , Zhang, Y. , Liu, J. , Gao, C. and Qiu, J.L. (2014) Simultaneous editing of three homoeoalleles in hexaploid bread wheat confers heritable resistance to powdery mildew. Nat. Biotechnol. 32, 947–951. 25038773 10.1038/nbt.2969

[pbi12611-bib-0033] Wang, S. , Zhang, S. , Wang, W. , Xiong, X. , Meng, F. and Cui, X. (2015) Efficient targeted mutagenesis in potato by the CRISPR/Cas9 system. Plant Cell Rep. 34, 1473–1476. 26082432 10.1007/s00299-015-1816-7

[pbi12611-bib-0034] Woo, J.W. , Kim, J. , Kwon, S.I. , Corvalan, C. , Cho, S.W. , Kim, H. , Kim, S.G. , *et al*. (2015) DNA‐free genome editing in plants with preassembled CRISPR‐Cas9 ribonucleoproteins. Nat. Biotechnol. 33, 1162–1164. 26479191 10.1038/nbt.3389

[pbi12611-bib-0035] Wu, X. , Scott, D.A. , Kriz, A.J. , Chiu, A.C. , Hsu, P.D. , Dadon, D.B. , Cheng, A.W. , *et al*. (2014) Genome‐wide binding of the CRISPR endonuclease Cas9 in mammalian cells. Nat. Biotechnol. 32, 670–676. 24752079 10.1038/nbt.2889 PMC4145672

[pbi12611-bib-0036] Xie, K. , Minkenberg, B. and Yang, Y. (2015) Boosting CRISPR/Cas9 multiplex editing capability with the endogenous tRNA‐processing system. Proc. Natl Acad. Sci. USA, 112, 3570–3575. 25733849 10.1073/pnas.1420294112 PMC4371917

[pbi12611-bib-0037] Xing, H.L. , Dong, L. , Wang, Z.P. , Zhang, H.Y. , Han, C.Y. , Liu, B. , Wang, X.C. , *et al*. (2014) A CRISPR/Cas9 toolkit for multiplex genome editing in plants. BMC Plant Biol. 14, 327. 25432517 10.1186/s12870-014-0327-y PMC4262988

[pbi12611-bib-0038] Xu, K. , Ren, C. , Liu, Z. , Zhang, T. , Zhang, T. , Li, D. , Wang, L. , *et al*. (2015) Efficient genome engineering in eukaryotes using Cas9 from *Streptococcus thermophilus* . Cell. Mol. Life Sci. 72, 383–399. 25038777 10.1007/s00018-014-1679-z PMC11113816

[pbi12611-bib-0039] Yuan, T. , Fujioka, S. , Takatsuto, S. , Matsumoto, S. , Gou, X. , He, K. , Russell, S.D. , *et al*. (2007) Ben1, a gene encoding a dihydroflavonol 4‐reductase (DFR)‐like protein, regulates the levels of brassinosteroids in *Arabidopsis thaliana* . Plant J. 51, 220–233. 17521414 10.1111/j.1365-313X.2007.03129.x

[pbi12611-bib-0040] Zhai, J. , Zhang, H. , Arikit, S. , Huang, K. , Nan, G.L. , Walbot, V. and Meyers, B.C. (2015) Spatiotemporally dynamic, cell‐type‐dependent premeiotic and meiotic phasiRNAs in maize anthers. Proc. Natl Acad. Sci. USA 112, 3146–3151. 25713378 10.1073/pnas.1418918112 PMC4364226

[pbi12611-bib-0041] Zhang, H. , Zhang, J. , Wei, P. , Zhang, B. , Gou, F. , Feng, Z. , Mao, Y. , *et al*. (2014) The CRISPR/Cas9 system produces specific and homozygous targeted gene editing in rice in one generation. Plant Biotechnol. J. 12, 797–807. 24854982 10.1111/pbi.12200

[pbi12611-bib-0042] Zhou, H. , Liu, B. , Weeks, D.P. , Spalding, M.H. and Yang, B. (2014) Large chromosomal deletions and heritable small genetic changes induced by CRISPR/Cas9 in rice. Nucleic Acids Res. 42, 10903–10914. 25200087 10.1093/nar/gku806 PMC4176183

[pbi12611-bib-0043] Zhu, J. , Song, N. , Sun, S. , Yang, W. , Zhao, H. , Song, W. and Lai, J. (2016) Efficiency and inheritance of targeted mutagenesis in maize using CRISPR‐Cas9. J. Genet. Genom. 43, 25–36. 10.1016/j.jgg.2015.10.006 26842991

