# Supplementary material for: An Agrobacterium‐delivered CRISPR/Cas9 system for high‐frequency targeted mutagenesis in maize
Source: Plant Biotechnol J. 2016 Sep 5;15(2):257–68. doi: 10.1111/pbi.12611 (PMC5259581; doi:10.1111/pbi.12611)
Supplement: Supplementary file 1 — Figure S1 a1 and a4 Genotypes of CRISPR line 24‐3. Table S1 Primers and sequences used in this study. [file PBI-15-257-s001.pdf]

SUPPORTING INFORMATION

Figure S1. *a1* and *a4* Genotypes of CRISPR line 24-3.

Table S1. Primers and sequences used in this study.

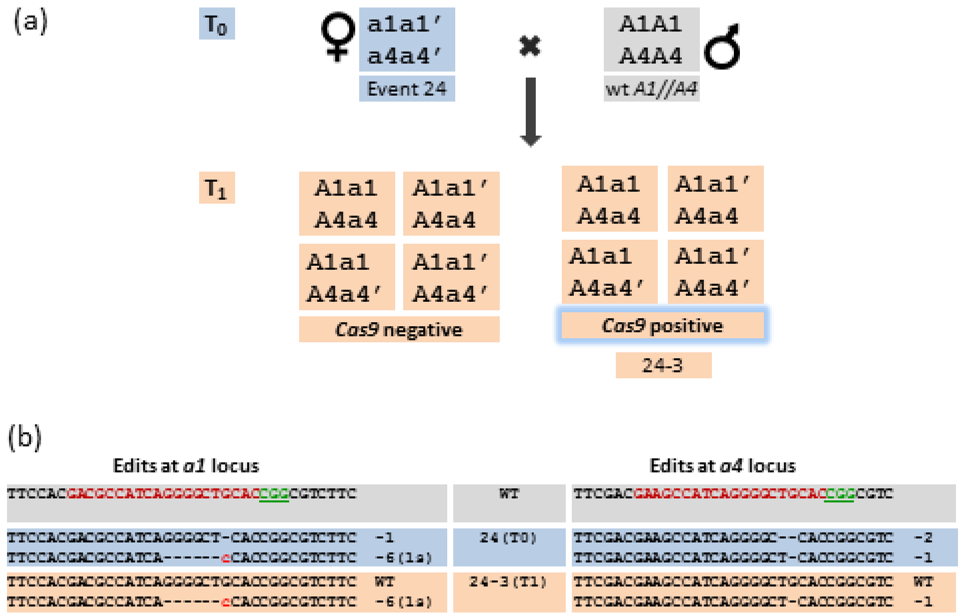

Figure S1. *a1* and *a4* genotype RT-PCR analysis of CRISPR line 24-3, a Cas9/gRNA line with no detectable *Cas9* gene expression (Figure 5b). (a) Schematic diagram shows the transmission genetic analysis of edited alleles from the T_0 plant and the T_1 progeny of event 24. The T_0 line has DA mutations for both *A1* and *A4*, a total of four mutations. The T_1 progeny were derived by crossing mutants to recipient lines with wild-type *A1* and *A4* loci. The wild type allele is represented as ‘A’, T_0 edited alleles as ‘a’ and ‘a'’. (b) Sequence information at the *a1* and *a4* targeted loci for the T_0, and T_1 plants of event 24-3. Nucleotides in red represent target sites and green/underlined indicate PAM sequences for the gRNAs. The nucleotide variations (dashes for deletion and lower case letter in blue for insertion) are marked on the right side of each sequence with a number, suffixed with a letter, if needed, to distinguish different alleles. Line names are listed in the middle column.

Supplemental Table 1. Primers and sequences used in this study

Primers	Sequences (5' to 3')	Purpose
gAGO18a-F1	TGTTGAGAGCTGCCCTTCAAGACCA	Construct gAGO18a under U6.1
gAGO18a-R1	AAACTGGTCTTGAAGGGCAGCTCTC	Construct gAGO18a under U6.1
gAGO18a-F2	GTGTGCCTTGTCTGGCAGGATGGAA	Construct gAGO18a under U6.2
gAGO18a-R2	AAACTTCCATCCTGCCAGACAAGGC	Construct gAGO18a under U6.2
gAGO18b-F1	TGTTGACTAAACCTGAGTATGACA	Construct gAGO18b under U6.1
gAGO18b-R1	AAACTGTCATACTCAGGTTTAGTC	Construct gAGO18b under U6.1
gAGO18b-F2	GTGTGTTCAGCTCTTGAAGGCCCTC	Construct gAGO18b under U6.2
gAGO18b-R2	AAACGAGGGCCTTCAAGAGCTGAAC	Construct gAGO18b under U6.2
Ago18a-F1	GTCGTCAAGGCCAACCACTTC	Genotype *ZmAGO18a* locus
Ago18a-R1	CATGAGCATCAACAGCTGGTC	Genotype *ZmAGO18a* locus
Ago18b-F1	ATGTGGTTGGTGACTTTGTAC	Genotype *ZmAGO18b* locus
Ago18b-R1	AGAACAATCCTACCTCAAATC	Genotype *ZmAGO18b* locus
gBEN1-F1	TGTTGAAGCCATCAGGGGCTGCAC	Construct gBEN1 under U6.1
gBEN1-R1	AAACGTGCAGCCCCTGATGGCTTC	Construct gBEN1 under U6.1
gBEN1-F2	GTGTGTGATCAAGCCGACGGTGGA	Construct gBEN1 under U6.2
gBEN1-R2	AAACTCCACCGTCGGCTTGATCAC	Construct gBEN1 under U6.2
BEN1-a4-F1	ATCCAAACCCGTAGCAGCAGA	Genotype *BEN1-a4* locus
BEN1-a4-R1	AGCGACGAAGTTCGGTTGAATTG	Genotype *BEN1-a4* locus
BEN1-a1-F1	AGCCAAACTCTGATTCGCTCC	Genotype *BEN1-a1* locus
BEN1-a1-R1	CGTCGAAGTTCAGTTGAATTG	Genotype *BEN1-a1* locus
OsCas9-F	GGGTAATGAACTCGCTCTGC	Genotype null segregant
OsCas9-R	TGGCGTCAAGAACTTCCTTTG	Genotype null segregant
BarXho-F	CCCTCGAGTCTACCATGAGCCCAGAACGAC	Genotype null segregant
BarXho-R	CCCTCGAGTCAAATCTCGGTGACGGGCAGGAC	Genotype null segregant
U6P-F1b	CGTTGAGGGGAGACAGGTTTAG	PCR and sequencing primer of gRNA genes
pENTR4-R	TGGGTCTAGATATCTCGAGTG	PCR and sequencing primers of gRNA genes
Ubi-F	TAAGCTGCCGATGTGCCTGCGTCG	RT-PCR
Ubi-R	CTGAAAGACAGAACATAATGAGCACAG	RT-PCR
